# Predicting Long COVID in the National COVID Cohort Collaborative Using Super Learner: Cohort Study

**DOI:** 10.2196/53322

**Published:** 2024-08-15

**Authors:** Zachary Butzin-Dozier, Yunwen Ji, Haodong Li, Jeremy Coyle, Junming Shi, Rachael V Phillips, Andrew N Mertens, Romain Pirracchio, Mark J van der Laan, Rena C Patel, John M Colford, Alan E Hubbard, The National COVID Cohort Collaborative (N3C) Consortium

**Affiliations:** 1 Division of Biostatistics University of California Berkeley School of Public Health Berkeley, CA United States; 2 Department of Anesthesia and Perioperative Care University of California San Francisco San Francisco, CA United States; 3 Department of Infectious Diseases University of Alabama at Birmingham School of Medicine Birmingham, AL United States; 4 Members are listed at the end of the manuscript

**Keywords:** long COVID, COVID-19, machine learning, respiratory, infectious, SARS-CoV-2, sequelae, chronic, long term, covariate, covariates, risk, risks, predict, prediction, predictions, predictive, Super Learner, ensemble, stacking

## Abstract

**Background:**

Postacute sequelae of COVID-19 (PASC), also known as long COVID, is a broad grouping of a range of long-term symptoms following acute COVID-19. These symptoms can occur across a range of biological systems, leading to challenges in determining risk factors for PASC and the causal etiology of this disorder. An understanding of characteristics that are predictive of future PASC is valuable, as this can inform the identification of high-risk individuals and future preventative efforts. However, current knowledge regarding PASC risk factors is limited.

**Objective:**

Using a sample of 55,257 patients (at a ratio of 1 patient with PASC to 4 matched controls) from the National COVID Cohort Collaborative, as part of the National Institutes of Health Long COVID Computational Challenge, we sought to predict individual risk of PASC diagnosis from a curated set of clinically informed covariates. The National COVID Cohort Collaborative includes electronic health records for more than 22 million patients from 84 sites across the United States.

**Methods:**

We predicted individual PASC status, given covariate information, using Super Learner (an ensemble machine learning algorithm also known as stacking) to learn the optimal combination of gradient boosting and random forest algorithms to maximize the area under the receiver operator curve. We evaluated variable importance (Shapley values) based on 3 levels: individual features, temporal windows, and clinical domains. We externally validated these findings using a holdout set of randomly selected study sites.

**Results:**

We were able to predict individual PASC diagnoses accurately (area under the curve 0.874). The individual features of the length of observation period, number of health care interactions during acute COVID-19, and viral lower respiratory infection were the most predictive of subsequent PASC diagnosis. Temporally, we found that baseline characteristics were the most predictive of future PASC diagnosis, compared with characteristics immediately before, during, or after acute COVID-19. We found that the clinical domains of health care use, demographics or anthropometry, and respiratory factors were the most predictive of PASC diagnosis.

**Conclusions:**

The methods outlined here provide an open-source, applied example of using Super Learner to predict PASC status using electronic health record data, which can be replicated across a variety of settings. Across individual predictors and clinical domains, we consistently found that factors related to health care use were the strongest predictors of PASC diagnosis. This indicates that any observational studies using PASC diagnosis as a primary outcome must rigorously account for heterogeneous health care use. Our temporal findings support the hypothesis that clinicians may be able to accurately assess the risk of PASC in patients before acute COVID-19 diagnosis, which could improve early interventions and preventive care. Our findings also highlight the importance of respiratory characteristics in PASC risk assessment.

**International Registered Report Identifier (IRRID):**

RR2-10.1101/2023.07.27.23293272

## Introduction

As the mortality rate associated with acute COVID-19 incidence wanes, investigators have shifted focus to determining its longer-term, chronic impacts [1]. Postacute sequelae of COVID-19 (PASC), also known as long COVID, is a loosely categorized consequence of acute infection that is related to dysfunction across multiple biological systems [2]. Much remains unknown about PASC, leaving individuals uncertain regarding their risk for PASC and what factors may contribute to this risk. Prediction of individual risk for PASC diagnosis can allow us to identify what populations are at the greatest risk for PASC, and interpretation of these predictors may generate hypotheses regarding underlying drivers of PASC incidence.

Electronic health record (EHR) databases, such as the National COVID Cohort Collaborative (N3C), provide an important tool for predicting, evaluating, and understanding PASC [3,4]. There is a broad range of PASC symptoms, diagnostic criteria, and hypothesized causal mechanisms, which has made it difficult for investigators to build generalizable predictions (Multimedia Appendix 1) [5-7]. Given this heterogeneity, multisite evaluations including large sample sizes and high-dimensional covariate information can provide opportunities to build models that can accurately predict PASC risk.

Due to the broad range of factors associated with PASC, the high dimensionality of the large EHR databases, and the unknown determinants of PASC, modeling methods for predicting PASC must be highly flexible. Super Learner (SL) is a flexible, ensemble (stacked) machine learning algorithm that uses cross-validation to learn the optimal weighted combination of a specified set of algorithms [8,9]. The SL is grounded in statistical optimality theory that guarantees it will perform at least as well as the best-performing algorithm included in the library for large sample sizes. Thus, a rich library of learners, with a sufficient sample size, will ensure optimal performance. The SL can be specified to maximize any performance metric, such as mean squared error [9]. Given the large sample size of high-dimensional data in EHR databases, SL is well positioned to predict individual risk of PASC diagnosis in this setting.

Here, we used the SL to predict PASC diagnosis in patients with COVID-19, given a diverse set of features curated from the EHR. We also investigated the importance of features for predicting PASC by assessing the importance of each individual feature, by assessing groups of features based on temporality (baseline, pre–COVID-19, acute COVID, and post–COVID-19 features) and by hypothesized clinical domains of PASC.

## Methods

### Sample

The Long COVID Computational Challenge (DUR RP-5A73BA) sample population was selected from the N3C data set, a national, open data set that has been described previously [3,4]. N3C has created a centralized repository where investigators can access and analyze data from more than 8 million patients with COVID-19, including 32 billion rows of data from 84 sites across the United States while maintaining patient privacy [10,11]. When a patient at a participating site is diagnosed with COVID-19, they are included in the N3C database, along with 2 sociodemographically matched controls. N3C defines acute COVID-19 diagnosis as either (1) at least 1 laboratory diagnostic positive result (either PCR or antigen) or (2) a provider diagnosis (*International Classification of Diseases, 10th Revision, Clinical Modification* [*ICD-10-CM*] code U07.1). We defined the index COVID-19 date as the earliest of these 2 dates [12,13]. For each sampled patient, N3C includes EHRs from January 1, 2018, to the present. These records include extensive information related to comorbidities, medications, medical procedures, demographic information, anthropometry, and other information collected during health care interactions.

The Long COVID Computational Challenge sample included cases of patients diagnosed with PASC (International Classification of Diseases [ICD] code U09.9) and matched controls with a documented COVID-19 diagnosis who had at least 1 health care interaction more than 4 weeks after their initial COVID-19 diagnosis date. ICD code U09.9, which was established on October 1, 2022, indicates a diagnosis for reimbursement purposes and enables linkage with COVID-19 diagnosis for patients experiencing postacute sequelae of infection [14]. Controls were selected at a 1:4 (case:control) ratio and were matched based on the distribution of health care interactions before a COVID-19 diagnosis. The primary outcome of interest was PASC diagnosis via ICD code U09.9. To evaluate our model’s discriminative ability, we used a 10% holdout test set based on study site (contributing data partner). In comparison to choosing a holdout test set randomly, nonrandom selection by factors such as study site improves the external validity of our model, as it evaluates the model’s predictive performance using data from a separate source [15]. We included data from the beginning of the N3C observation period (January 1, 2018) to 28 days following acute COVID-19.

### Feature Selection

#### Overview

Our set of features for predicting PASC included those previously described in the literature [3] and additional features related to subject-matter knowledge and patterns of missingness. We extracted 304 features from N3C data. After indexing across 4 time periods (detailed below) and transforming features into formats amenable to machine learning analysis, our sample included 1339 features (see Multimedia Appendix 2). Details regarding feature selection and processing can be accessed via GitHub [16]. For continuous features, we included the minimum, maximum, and mean values for each measurement in each temporal window. For binary features, we either included an indicator (when repetition was not relevant) or a count (when repetition was relevant) over each time period, and we recoded categorical variables as indicators.

#### Temporal Windows

We divided each participant’s records into 4 temporal windows: baseline, which consisted of all records occurring a minimum of 37 days before the COVID-19 index date (*t* – 37, where *t* represents the COVID-19 index date), and all time-invariant factors (such as sex, ethnicity, etc); pre–COVID-19, observations falling between 37 and 7 days before the index date (*t* – 37 to *t* – 7); acute COVID-19, observations falling 7 days before 14 days after to the index date (*t* – 7 to *t* + 14); and post–COVID-19, records from 14 to 28 days after the index date (*t* + 14 to *t* + 28). The acute COVID-19 window begins 7 days before the reported infection date, to conservatively include early COVID-19 symptoms before official diagnosis.

#### Features Described in the Literature

We extracted and transformed key features that were identified in prior research using N3C data as risk factors for PASC [3]. These features included 199 previously described factors related to medical history, diagnoses, demographics, and comorbidities [3].

#### Temporality

To account for differences in follow-up, we included as an additional factor a continuous variable for follow-up time, defined as the number of days between the COVID-19 index date and the most recent observation. To account for temporal trends of COVID-19 (such as seasonality and dominant variant), we included categorical (ordinal) covariates for the season and months since the first observed COVID-19 index date.

#### Missing Data

To avoid dropping any observations, we mean-imputed missing observations for continuous variables and added indicator variables for imputed values [17]. By using flexible ensemble machine learning, which allows for interactions between imputed variables and the missingness indicators, we allow the patterns of missingness to be potential predictors of PASC. Furthermore, as SL predicts the outcome based on a semiparametric function of all predictor variables, including missingness, this workflow implicitly imputes missing variables using the candidate algorithms in the SL. Therefore, further imputation of missing predictor values is not necessary.

#### COVID-19 Positivity

We added several measures of COVID-19 severity and persistent SARS-CoV-2 viral load, which are associated with PASC incidence [18]. We imported measures of COVID-19 severity as well as 15 measures of acute COVID-19 from laboratory measurements, which provided insights into persistent SARS-CoV-2 viral load. We assessed the duration of COVID-19 viral positivity separately for each laboratory measure of COVID-19 and each temporal window. For participants who had both a positive and negative value of a given test during a temporal window, we took the midpoint between the last positive test and the first negative test as being the end point of their positivity. For individuals who had a positive test but no subsequent negative test within that temporal window, we determined their end point to be their final positive test plus 3 days. We included separate missingness indicators in each temporal window for each test, for a positive value for each test, and for a negative value following a positive value to indicate an imputed positivity end point. We included the calendar date of index infection to account for the COVID-19 viral strain, given our lack of variant data.

#### Additional Features

We incorporated the laboratory measurements related to anthropometry, nutrition, COVID-19 positivity, inflammation, tissue damage due to viral infection, autoantibodies and immunity, cardiovascular health, and microvascular disease, which are potential predictors of PASC [18]. We also extracted information about smoking status, alcohol use, marital status, and use of insulin or anticoagulant from the observation table as baseline characteristics of individuals. We included the number of times a person has been exposed to respiratory devices (eg, supplemental oxygen or ventilator) in each of the 4 windows from the device table. We extracted covariates related to COVID-19 severity, vaccination history, demographics, medical history, and previous diagnoses from before and during acute COVID-19.

### Prediction Using Ensemble Machine Learning

We used the SL, an ensemble machine learning method, also known as stacking, to learn the optimally weighted combination of candidate algorithms for maximizing the area under the curve (AUC). We reprogrammed the SL in Python to capitalize on the resources (eg, PySpark parallelization) available in the N3C Data Enclave (National Center for Advancing Translational Sciences; NCATS), and this software is available to external researchers [16]. We used an ensemble of 4 learners (a mix of parametric models and machine learning models): (1) logistic regression, (2) L1 penalized logistic regression (with penalty parameter lambda=0.01), (3) gradient boosting (with n_estimators=200, max_depth=5, and learning_rate=0.1), and (4) random forest (with max_depth=5 and num_trees=20). The original candidate learner library consisted of a large set of candidate learners with different combinations of hyperparameters (eg, gradient boosting with n_estimators=[.200, 150, 100, 50], max_depth=[.3, 5, 7], and learning_rate=[.0.05, 0.1, 0.2]). SL is based on the Oracle Inequality, and there is strong theoretical justification for its use of k-fold cross-validation [19]. Modifications to this approach, such as repeated cross-validation, may provide a benefit in finite sample situations, but given the large sample size available here in N3C, these modifications would have little impact on performance in this context [8,9,19].

To tune the hyperparameters for the candidate algorithms, first, we randomly split the full data into a training set (with 0.9 of the sample) and a test set (0.1 of the sample). Then, we prespecified a grid of candidate values for hyperparameters including a maximum number of iterations, learning rate, maximum depth of trees, and feature subset strategy. Then, we fit the algorithms with these candidate values on the training set and collected the loss on the test set. Finally, for each hyperparameter of each algorithm, we plotted the training and testing errors against the candidate values and select the ones where the testing errors stop decreasing. We then equipped the algorithms with the best hyperparameter candidate and include them in the SL library. Without computational constraints, one can treat each algorithm with unique hyperparameter values as separate candidate learners in the library, which can incorporate automated hyperparameter tuning as part of the SL process. In this project, we separated the tuning part from the model fitting process due to the computational constraints of the N3C enclave.

### Prediction Performance

One important decision for optimizing an algorithm is to choose the metric that will be used to evaluate the fit and optimize the weighting of the algorithms in the ensemble. We used an approach developed specifically for maximizing the AUC [20]. The SL was specified such that it learned the combination of algorithms, including variations of gradient boosting (extreme gradient boosting) and random forest, that maximized the AUC [20]. Specifically, we used an AUC maximizing meta-learner with Powell optimization to learn the convex combination of these 4 candidate algorithms [20]. The SL was implemented with a V-fold or k-fold cross-validation scheme with 10 folds. To evaluate model performance, we reported the AUC, accuracy, precision, recall, *F*_1_-score, and Brier score, along with associated 95% CIs, for our ensemble algorithm [21].

### Variable Importance

For the sake of computational efficiency, we worked with the discrete SL selector (the single candidate learner in the library with the highest cross-validated AUC) instead of the entire ensemble SL. In this case, the gradient-boosting learner was the candidate learner with the highest cross-validated AUC. As the gradient-boosting algorithm carried the vast majority (75%) of the weight of the ensemble SL, the variable importance of this algorithm is an appropriate summary of the overall ensemble. We used a general approach (for any machine learning algorithm) known as Shapley values [22]. We generated these values within 3 groupings of predictors for ease of interpretability: individual features (eg, cough diagnosis during acute COVID-19 window), the temporal window when measurements were made relative to acute COVID-19 (eg, pre–COVID-19 window), and specific clinical domains (eg, respiratory pathway). At the individual level, we assessed the importance of each variable (indexed across each of the 4 temporal windows) in predicting PASC. At the temporal level, we assessed the relative importance of each of the 4 temporal windows (baseline, pre–COVID-19, acute COVID-19, and post–COVID-19) in predicting PASC status. At the level of the clinical domain, we grouped variables based on the following hypothesized mechanistic pathways of PASC: (1) baseline demographics and anthropometry; (2) health care use; (3) respiratory system; (4) antimicrobials and infectious disease; (5) cardiovascular system; (6) female hormones and pregnancy; (7) mental health and well-being; (8) pain, skin sensitivity, and headaches; (9) digestive system; (10) inflammation, autoimmune, and autoantibodies; (11) renal function, liver function, and diabetes; (12) nutrition; (13) COVID-19 positivity; and (14) uncategorized disease, nervous system, injury, mobility, and age-related factors [18]. For temporal and clinical domain groupings, we assessed the mean Shapley value of the 10 most predictive features in each group. A full list of our included covariates along with their grouping by temporality and clinical domain is included in Multimedia Appendix 2.

### Ethical Considerations

The N3C data transfer to NCATS is performed under a Johns Hopkins University Reliance Protocol (IRB00249128) or individual site agreements with the National Institutes of Health (NIH). The N3C Data Enclave is managed under the authority of the NIH; information can be found online [23]. This study was approved by the University of California Berkeley Office for Protection of Human Subjects (2022-01-14980). N3C received a waiver of consent from the NIH institutional review board and allows the secondary analysis of these data without additional consent. NCATS ensures that the privacy of patient data is maintained by managing access to the N3C data enclave, the use of patient data in the N3C Enclave, and the publication of inferences drawn from these data. Patients were not compensated for this research.

## Results

### Overview

The data set included 57,672 patients with 9031 cases; 46,226 controls; and 2415 patients excluded due to having a PASC diagnosis within 4 weeks of an acute COVID-19 diagnosis. This yielded a final analytic sample of 55,257 participants (Table 1).

**Table 1:** Characteristics of the sample population. The sample population was drawn from electronic health record data of patients included in the National COVID Cohort Collaborative during the COVID-19 pandemic (N=55,257).

Characteristics		Values
**Sex, n (%)**		
	Female	32,534 (58.88)
	Male	22,675 (41.04)
	Unknown	48 (0.09)
**Race, n (%)**		
	Asian or Pacific Islander	1303 (2.36)
	Black or African American	11,481 (20.78)
	White	32,411 (58.66)
	Other	1087 (1.97)
	Unknown	8975 (16.24)
**Ethnicity, n (%)**		
	Not Hispanic or Latino	43,282 (78.33)
	Hispanic or Latino	5363 (9.71)
	Unknown	6612 (11.97)
**Age (years)**		
	<18, n (%)	6393 (11.57)
	18-25, n (%)	5021 (9.09)
	26-45, n (%)	15,660 (28.34)
	46-65, n (%)	15,291 (27.67)
	≥66, n (%)	8153 (14.75)
	Mean (SD)	43.33 (20.71)
**Pre–COVID-19 comorbidities, n (%)**		
	Diabetes	5623 (10.18)
	Chronic kidney disease	2835 (5.13)
	Congestive heart failure	2396 (4.34)
	Chronic pulmonary disease	696 (1.26)
**COVID-19 severity type, n (%)**		
	Mild (no emergency visit)	47,351 (85.69)
	Mild (with emergency visit)	3159 (5.72)
	Moderate (with hospitalization)	3914 (7.08)
	Severe (with extracorporeal membrane oxygenation or invasive mechanical ventilation)	720 (1.30)
	Death following infection	104 (0.19)
**BMI, n (%)**		
	Obese	4556 (8.25)
	Severely obese	2798 (5.06)

### Predictive Performance

**Figure 1:**
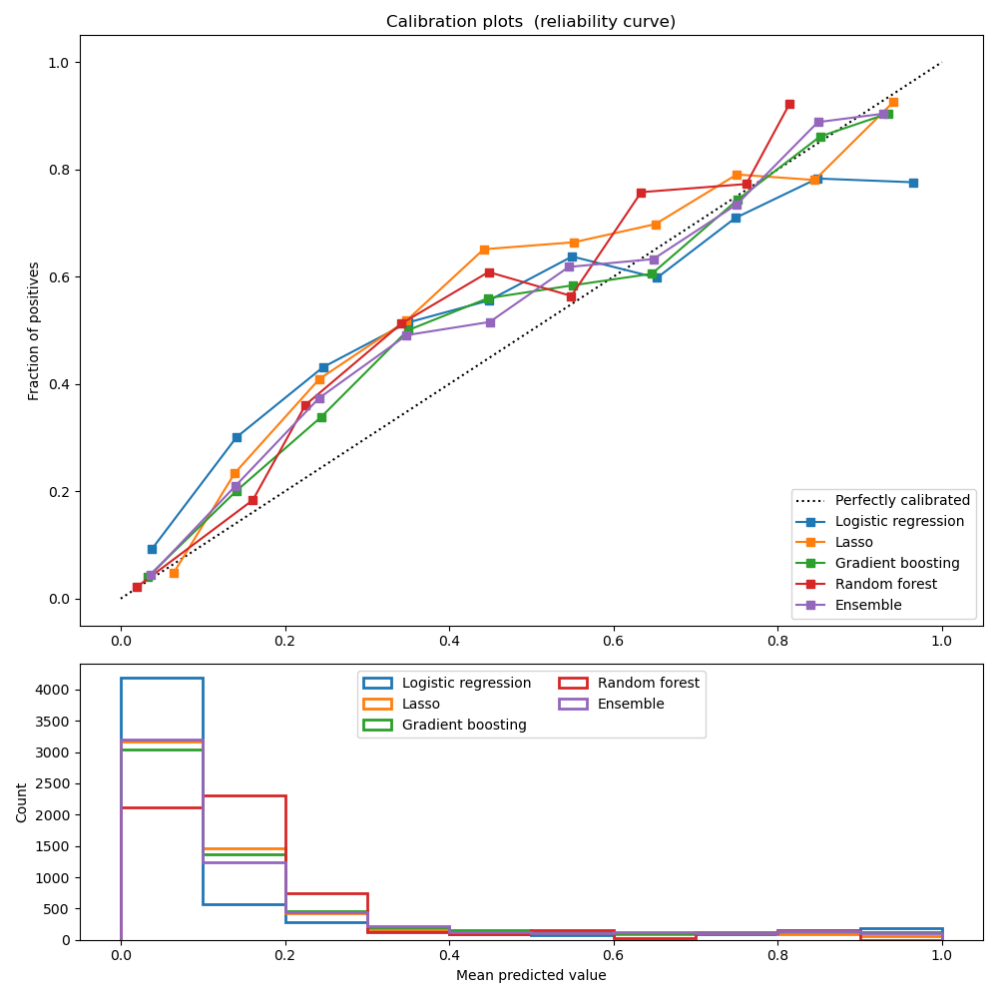
Calibration of candidate learners and the ensemble algorithm in PASC diagnosis. Model created using electronic health record data from a sample of patients included in the National COVID Cohort Collaborative during the COVID-19 pandemic. PASC: postacute sequelae of COVID-19.

Our ensemble machine learning algorithm achieved an AUC of 0.874 (95% CI 0.864-0.884), accuracy of 0.772 (95% CI 0.761-0.783), precision of 0.467 (95% CI 0.446-0.489), recall of 0.806 (95% CI 0.784-0.828), *F*_1_-score of 0.591 (95% CI 0.571-0.661), and Brier score of 0.110 (95% CI 0.104-0.116; see Table 2). We report the calibration metrics for each candidate algorithm (logistic regression, Lasso, gradient boosting, and random forest) and the ensemble algorithm in Figure 1. All models slightly underestimate the sample patient risk of PASC diagnosis over this study’s period.

**Table 2:** Performance of the ensemble Super Learner in the prediction of postacute sequelae of COVID-19 diagnosis. Model created using electronic health record data from a sample of patients included in the National COVID Cohort Collaborative during the COVID-19 pandemic.

Metric	Estimate (95% CI)
Area under the receiver operator curve	0.874 (0.864-0.884)
Accuracy	0.772 (0.761-0.783)
Precision	0.467 (0.446-0.489)
Recall	0.806 (0.784-0.828)
*F*_1_-score	0.591 (0.571-0.661)
Brier score	0.110 (0.104-0.116)

### Variable Importance

#### Individual Predictors

We found that the strongest individual predictors (absolute Shapley value) of PASC diagnosis were observation period length (0.075), the number of health care interactions associated with a diagnosis during the acute COVID-19 period (0.045), viral lower respiratory infection during the acute COVID-19 period (0.040), age (0.038), ethnicity unknown (0.037), total number of health care interactions associated with a diagnosis during the post–COVID-19 period (0.035), systemic corticosteroid use before acute COVID-19 (0.025), index acute COVID-19 date (0.024), state missing (0.019), state Utah (0.016), state Ohio (0.016), acute respiratory disease during the acute COVID-19 period (0.013), creatinine concentration in blood (0.012), never smoker (0.011), total health care interactions associated with a diagnosis during the baseline period (0.011), cough during the acute COVID-19 period (0.011), cough during the post–COVID-19 period (0.011), state Colorado (0.010), creatinine volume (0.010), and SARS-CoV-2 RNA presence (0.010; Table 3).

**Table 3:** Most important model features associated with postacute sequelae of COVID-19 ranked by absolute Shapley value. Model created using electronic health record data from a sample of patients included in the National COVID Cohort Collaborative during the COVID-19 pandemic. For additional information regarding covariates, see Multimedia Appendix 2.

Feature	Absolute Shapley value, mean
Observation period length	0.075
Number of health care interactions during the acute COVID-19 period	0.045
Viral lower respiratory infection during the acute COVID-19 period	0.040
Age (years)	0.038
Ethnicity unknown	0.037
Total number of health care interactions associated with a diagnosis during the post–COVID-19 period	0.035
Systemic corticosteroid use before acute COVID-19	0.025
Index acute COVID-19 date	0.024
State missing	0.019
State Utah	0.016
State Ohio	0.016
Acute respiratory disease during the acute COVID-19 period	0.013
Creatinine concentration in blood	0.012
Never smoker	0.011
Total health care interactions associated with a diagnosis during the baseline period	0.011
Cough during the acute COVID-19 period	0.011
Cough during the post–COVID-19 period	0.011
State Colorado	0.010
Creatinine volume	0.010
SARS-CoV-2 RNA presence	0.010

#### Temporal windows

Baseline and time-invariant characteristics were the strongest predictors of PASC (mean 0.026, SD 0.020), followed by characteristics during the acute COVID-19 period (mean 0.016, SD 0.015), the post–COVID-19 period (mean 0.008, SD 0.010), and the pre–COVID-19 period (mean 0.006, SD 0.003; Table 4).

**Table 4:** Variable importance of features associated with postacute sequelae of COVID-19 diagnosis by the temporal window. Ranked by the mean absolute Shapley value of the top 10 features in each category. Model created using electronic health record data from a sample of patients included in the National COVID Cohort Collaborative during the COVID-19 pandemic. The temporal windows included baseline (before t – 37), pre–COVID-19 (t – 37 to t – 7), acute COVID-19 (t – 7 to t + 14), and post–COVID-19 (t + 14 to t + 28) periods, with t being the index COVID-19 date.

Temporal window	Absolute Shapley value of top 10 features, mean (SD)
Baseline	0.026 (0.020)
Acute COVID-19	0.016 (0.015)
Post–COVID-19	0.008 (0.010)
Pre–COVID-19	0.006 (0.003)

#### Clinical Domain

We found that health care interactions and procedures included the strongest predictors (mean 0.021, SD 0.024), followed by demographics and anthropometry (mean 0.016, SD 0.012), respiratory factors (mean 0.011, SD 0.011), renal and liver factors (mean 0.006, SD 0.004), cardiovascular factors (mean 0.005, SD 0.002), markers of COVID-19 positivity (mean 0.004, SD 0.003), inflammation markers (mean 0.003, SD 0.008), mental health factors (mean 0.002, SD 0.002), markers of pain (mean 0.002, SD 0.001), markers of nutrition (mean 0.001, SD 0.001), markers of general health and aging (mean 0.001, SD 0.000), factors related to female health and hormones (mean 0.001, SD 0.001), digestive health (mean 0.001, SD 0.001), and markers of general infectious disease (mean 0.001, SD 0.000; Table 5).

**Table 5:** Variable importance of features associated with postacute sequelae of COVID-19 diagnosis by the clinical domain. Ranked by the mean absolute Shapley value of the top 10 features (ranked by the same metric) in each category. Model created using electronic health record data from a sample of patients included in the National COVID Cohort Collaborative during the COVID-19 pandemic. For additional information regarding covariates, see Multimedia Appendix 2.

Clinical domain	Absolute Shapley value of top 10 features, mean (SD)
Health care interactions and procedures	0.021 (0.024)
Demographics and anthropometry	0.016 (0.012)
Respiratory factors	0.011 (0.011)
Renal and liver factors	0.006 (0.004)
Cardiovascular factors	0.005 (0.002)
Markers of COVID-19 positivity	0.004 (0.003)
Inflammation markers	0.003 (0.008)
Mental health factors	0.002 (0.002)
Markers of pain	0.002 (0.001)
Markers of nutrition	0.001 (0.001)
Markers of general health and aging	0.001 (0.000)
Factors related to female health and hormones	0.001 (0.001)
Digestive factors	0.001 (0.001)
Markers of general infectious disease	0.001 (0.000)

## Discussion

### Principal Findings

#### Overview

These results provide strong support for (1) the choice of an ensemble learning approach, (2) the specific learners used, (3) how the missing data were handled, and (4) the choice of optimization criteria (maximizing the AUC). These components are further supported by this model being awarded third place in the NIH Long COVID Computational Challenge (Multimedia Appendix 3 [2,3,8,18,20,22,24-29])*.* The findings of this study primarily serve to generate hypotheses for future investigation, although this ensemble model may provide utility in PASC risk assessment for patients following acute COVID-19 (as predictions were generated using data 4 weeks following acute infection).

#### Individual Predictors

We found that the individual predictors most associated with PASC diagnosis were related to health care use rate, such as observation period length and number of health care visits. These factors may not be causal drivers of PASC incidence and may, rather, indicate an incident diagnosis of PASC being more common among those already using medical care, which is consistent with the findings of Pfaff et al [3]. On the other hand, we found that lower tract viral respiratory infection during acute COVID-19 was highly predictive of PASC diagnosis. Previous studies have also linked lower respiratory infection during acute COVID-19 with negative outcomes. A 2022 study found that patients with COVID-19 with lower respiratory symptoms experienced worse health outcomes, including supplemental oxygen, mechanical ventilation, and death, compared to patients with upper respiratory symptoms or no respiratory symptoms [30]. Lower respiratory infection during acute COVID-19 may be a causal pathway by which acute COVID-19 leads to PASC, although future studies should apply a causal inference framework to evaluate this hypothesis.

#### Temporal Windows

We found that factors assessed during the baseline period (more than 37 days before COVID-19 diagnosis) were the strongest predictors of PASC diagnosis compared with factors immediately before, during, or after acute COVID-19. This suggests that clinicians may be able to effectively identify who is at risk for PASC based on baseline characteristics, such as preexisting conditions and sociodemographic information. Efforts to develop risk profiles based on these factors should be anchored within a social determinants of health approach, to reduce health inequity rather than reinforce systemic inequality [31]. However, it should be noted that baseline characteristics included the greatest interval of time and included time-variant factors, such as race. Future analyses should expand on this finding to evaluate the feasibility of predicting individual PASC incidence, rather than diagnosis (which may be subject to bias), using baseline characteristics alone. Additional information regarding this relationship could identify patients at risk for PASC before acute COVID-19 and could inform early interventions to prevent PASC.

#### Clinical Domain

These results are consistent with published literature and highlight the importance of respiratory features (eg, preexisting asthma) as important factors in predicting who may develop PASC [2,3]. Respiratory factors that may influence individual susceptibility to COVID-19 appear to be important features of acute COVID-19 severity and are key symptoms of PASC [2,3,25]. Therefore, future studies should seek to parse the contributions of respiratory symptoms to PASC through the pathways of baseline susceptibility to COVID-19 versus phenotyping of severe COVID-19 to improve our understanding of respiratory features as a risk factor for PASC. Despite the range of PASC phenotypes, these findings are consistent with respiratory symptoms (eg, dyspnea and cough) being the most commonly reported PASC symptoms [18,25]. Other clinical domains, such as cardiovascular factors, have similar roles as both markers of susceptibility and severity of COVID-19 and should also be explored further in future studies.

### Limitations

Our goal for this analysis was to maximize our model’s discriminative ability, rather than to make causal inferences regarding exposure-outcome relationships; therefore, we included all predictors before 4 weeks post–COVID-19 (censored window). First, the inclusion of pre–COVID-19, acute COVID, and post–COVID-19 factors complicate inference regarding whether predictive features (eg, respiratory factors) reflect vulnerability to acute COVID-19, COVID-19 symptoms, or early PASC symptoms. Second, this analytic sample was matched 1:4 (PASC:non-PASC), with matching based on pre–COVID-19 health care interaction rate, and this matched sample was drawn from N3C, which is a matched sample of patients with COVID-19 and healthy controls. Therefore, this sample may not be representative of a broader population. We note that, for future use of these data, if the prevalence of PASC in the target population is known, and the matching identifier is available, there are methods to calibrate the results to the actual population. Given that was not the case, one might generate results that need to be recalibrated to the target population of interest. Third, we found measures of health care use to be strong predictors of PASC diagnosis. It is plausible that health care use may be associated with increased diagnoses of various medical conditions in general, rather than true PASC incidence. However, increased health care use may also be an effect of early PASC symptoms. Finally, as is common with EHR data, N3C data are heterogeneous concerning certain outcomes, including biomarker data and PASC diagnosis. An SL-based approach seeks to account for this heterogeneity by modeling underlying patterns of missingness, but residual bias and confounding remain plausible. Overall, this approach enables investigators to make accurate predictions with minimal assumptions despite these data limitations. To improve upon the interpretation and clinical applications of these findings, future studies should apply a causal inference approach to evaluate the potential causal impact of individual predictors on the risk of PASC. These findings are temporally dependent, as the SARS-CoV-2 virus and the COVID-19 pandemic continue to evolve. Although our model explicitly incorporates temporal information, such as the date of infection, future analyses should retrain this publicly available model to optimize this prediction framework for contemporary viral dynamics (eg, geospatial disease trends) [16].

### Conclusions

These findings provide support for the use of an AUC-maximizing SL approach to predict PASC status using N3C data, which may have utility across other binary outcomes in EHR data. We found that baseline factors were most predictive of PASC diagnosis, which may support future efforts to identify high-risk individuals for preventive interventions or monitoring. These findings highlight the importance of respiratory symptoms, health care use, and age in predicting PASC incidence. Although further investigation is needed, our findings could support the referral of patients with COVID-19 with severe respiratory symptoms for subsequent PASC monitoring. In future work, we plan to investigate predictive performance when only baseline information is used as input to classify PASC, as this provides a practical implementation based on readily available clinical features that could identify participants at risk of PASC before COVID-19 diagnosis.

## Appendix

Multimedia Appendix 1 Hypothesized mechanisms and symptoms of postacute sequelae of COVID-19.

Multimedia Appendix 2 Table of electronic health record metadata.

Multimedia Appendix 3 Competition write-up PDF.

## Abbreviations

AUC: area under the curve
EHR: electronic health record
ICD: International Classification of Diseases
ICD-10-CM: International Classification of Diseases, 10th Revision, Clinical Modification
N3C: National COVID Cohort Collaborative
NCATS: National Center for Advancing Translational Sciences
NIH: National Institutes of Health
PASC: postacute sequelae of COVID-19
SL: Super Learner

## Data Availability

A synthetic version of the analytic data set and analytic code are available via GitHub [16]. The full, deidentified data can be accessed via the National COVID Cohort Collaborative (N3C) Data Enclave. Access to the N3C Data Enclave is managed by National Center for Advancing Translational Sciences (NCATS) [32]. Interested researchers must first complete a data use agreement, and next a data use request, to access the N3C Data Enclave. Once access is granted, the N3C data use committee must review and approve all use of data and the publication committee must approve all publications involving N3C data.

The following institutions whose data is released or pending:

Available: Advocate Health Care Network — UL1TR002389: The Institute for Translational Medicine (ITM) • Boston University Medical Campus — UL1TR001430: Boston University Clinical and Translational Science Institute • Brown University — U54GM115677: Advance Clinical Translational Research (Advance-CTR) • Carilion Clinic — UL1TR003015: iTHRIV Integrated Translational health Research Institute of Virginia • Charleston Area Medical Center — U54GM104942: West Virginia Clinical and Translational Science Institute (WVCTSI) • Children’s Hospital Colorado — UL1TR002535: Colorado Clinical and Translational Sciences Institute • Columbia University Irving Medical Center — UL1TR001873: Irving Institute for Clinical and Translational Research • Duke University — UL1TR002553: Duke Clinical and Translational Science Institute • George Washington Children’s Research Institute — UL1TR001876: Clinical and Translational Science Institute at Children’s National (CTSA-CN) • George Washington University — UL1TR001876: Clinical and Translational Science Institute at Children’s National (CTSA-CN) • Indiana University School of Medicine — UL1TR002529: Indiana Clinical and Translational Science Institute • Johns Hopkins University — UL1TR003098: Johns Hopkins Institute for Clinical and Translational Research • Loyola Medicine — Loyola University Medical Center • Loyola University Medical Center — UL1TR002389: The Institute for Translational Medicine (ITM) • Maine Medical Center — U54GM115516: Northern New England Clinical & Translational Research (NNE-CTR) Network • Massachusetts General Brigham — UL1TR002541: Harvard Catalyst • Mayo Clinic Rochester — UL1TR002377: Mayo Clinic Center for Clinical and Translational Science (CCaTS) • Medical University of South Carolina — UL1TR001450: South Carolina Clinical & Translational Research Institute (SCTR) • Montefiore Medical Center — UL1TR002556: Institute for Clinical and Translational Research at Einstein and Montefiore • Nemours — U54GM104941: Delaware CTR ACCEL Program • NorthShore University HealthSystem — UL1TR002389: The Institute for Translational Medicine (ITM) • Northwestern University at Chicago — UL1TR001422: Northwestern University Clinical and Translational Science Institute (NUCATS) • OCHIN — INV-018455: Bill and Melinda Gates Foundation grant to Sage Bionetworks • Oregon Health & Science University — UL1TR002369: Oregon Clinical and Translational Research Institute • Penn State Health Milton S. Hershey Medical Center — UL1TR002014: Penn State Clinical and Translational Science Institute • Rush University Medical Center — UL1TR002389: The Institute for Translational Medicine (ITM) • Rutgers, The State University of New Jersey — UL1TR003017: New Jersey Alliance for Clinical and Translational Science • Stony Brook University — U24TR002306 • The Ohio State University — UL1TR002733: Center for Clinical and Translational Science • The State University of New York at Buffalo — UL1TR001412: Clinical and Translational Science Institute • The University of Chicago — UL1TR002389: The Institute for Translational Medicine (ITM) • The University of Iowa — UL1TR002537: Institute for Clinical and Translational Science • The University of Miami Leonard M. Miller School of Medicine — UL1TR002736: University of Miami Clinical and Translational Science Institute • The University of Michigan at Ann Arbor — UL1TR002240: Michigan Institute for Clinical and Health Research • The University of Texas Health Science Center at Houston — UL1TR003167: Center for Clinical and Translational Sciences (CCTS) • The University of Texas Medical Branch at Galveston — UL1TR001439: The Institute for Translational Sciences • The University of Utah — UL1TR002538: Uhealth Center for Clinical and Translational Science • Tufts Medical Center — UL1TR002544: Tufts Clinical and Translational Science Institute • Tulane University — UL1TR003096: Center for Clinical and Translational Science • University Medical Center New Orleans — U54GM104940: Louisiana Clinical and Translational Science (LA CaTS) Center • University of Alabama at Birmingham — UL1TR003096: Center for Clinical and Translational Science • University of Arkansas for Medical Sciences — UL1TR003107: UAMS Translational Research Institute • University of Cincinnati — UL1TR001425: Center for Clinical and Translational Science and Training • University of Colorado Denver, Anschutz Medical Campus — UL1TR002535: Colorado Clinical and Translational Sciences Institute • University of Illinois at Chicago — UL1TR002003: UIC Center for Clinical and Translational Science • University of Kansas Medical Center — UL1TR002366: Frontiers: University of Kansas Clinical and Translational Science Institute • University of Kentucky — UL1TR001998: UK Center for Clinical and Translational Science • University of Massachusetts Medical School Worcester — UL1TR001453: The UMass Center for Clinical and Translational Science (UMCCTS) • University of Minnesota — UL1TR002494: Clinical and Translational Science Institute • University of Mississippi Medical Center — U54GM115428: Mississippi Center for Clinical and Translational Research (CCTR) • University of Nebraska Medical Center — U54GM115458: Great Plains IDeA-Clinical & Translational Research • University of North Carolina at Chapel Hill — UL1TR002489: North Carolina Translational and Clinical Science Institute • University of Oklahoma Health Sciences Center — U54GM104938: Oklahoma Clinical and Translational Science Institute (OCTSI) • University of Rochester — UL1TR002001: UR Clinical & Translational Science Institute • University of Southern California — UL1TR001855: The Southern California Clinical and Translational Science Institute (SC CTSI) • University of Vermont — U54GM115516: Northern New England Clinical & Translational Research (NNE-CTR) Network • University of Virginia — UL1TR003015: iTHRIV Integrated Translational health Research Institute of Virginia • University of Washington — UL1TR002319: Institute of Translational Health Sciences • University of Wisconsin-Madison — UL1TR002373: UW Institute for Clinical and Translational Research • Vanderbilt University Medical Center — UL1TR002243: Vanderbilt Institute for Clinical and Translational Research • Virginia Commonwealth University — UL1TR002649: C. Kenneth and Dianne Wright Center for Clinical and Translational Research • Wake Forest University Health Sciences — UL1TR001420: Wake Forest Clinical and Translational Science Institute • Washington University in St. Louis — UL1TR002345: Institute of Clinical and Translational Sciences • Weill Medical College of Cornell University — UL1TR002384: Weill Cornell Medicine Clinical and Translational Science Center • West Virginia University — U54GM104942: West Virginia Clinical and Translational Science Institute (WVCTSI)

Submitted: Icahn School of Medicine at Mount Sinai — UL1TR001433: ConduITS Institute for Translational Sciences • The University of Texas Health Science Center at Tyler — UL1TR003167: Center for Clinical and Translational Sciences (CCTS) • University of California, Davis — UL1TR001860: UCDavis Health Clinical and Translational Science Center • University of California, Irvine — UL1TR001414: The UC Irvine Institute for Clinical and Translational Science (ICTS) • University of California, Los Angeles — UL1TR001881: UCLA Clinical Translational Science Institute • University of California, San Diego — UL1TR001442: Altman Clinical and Translational Research Institute • University of California, San Francisco — UL1TR001872: UCSF Clinical and Translational Science Institute

Pending: Arkansas Children’s Hospital — UL1TR003107: UAMS Translational Research Institute • Baylor College of Medicine — None (Voluntary) • Children’s Hospital of Philadelphia — UL1TR001878: Institute for Translational Medicine and Therapeutics • Cincinnati Children’s Hospital Medical Center — UL1TR001425: Center for Clinical and Translational Science and Training • Emory University — UL1TR002378: Georgia Clinical and Translational Science Alliance • HonorHealth — None (Voluntary) • Loyola University Chicago — UL1TR002389: The Institute for Translational Medicine (ITM) • Medical College of Wisconsin — UL1TR001436: Clinical and Translational Science Institute of Southeast Wisconsin • MedStar Health Research Institute — UL1TR001409: The Georgetown-Howard Universities Center for Clinical and Translational Science (GHUCCTS) • MetroHealth — None (Voluntary) • Montana State University — U54GM115371: American Indian/Alaska Native CTR • NYU Langone Medical Center — UL1TR001445: Langone Health’s Clinical and Translational Science Institute • Ochsner Medical Center — U54GM104940: Louisiana Clinical and Translational Science (LA CaTS) Center • Regenstrief Institute — UL1TR002529: Indiana Clinical and Translational Science Institute • Sanford Research — None (Voluntary) • Stanford University — UL1TR003142: Spectrum: The Stanford Center for Clinical and Translational Research and Education • The Rockefeller University — UL1TR001866: Center for Clinical and Translational Science • The Scripps Research Institute — UL1TR002550: Scripps Research Translational Institute • University of Florida — UL1TR001427: UF Clinical and Translational Science Institute • University of New Mexico Health Sciences Center — UL1TR001449: University of New Mexico Clinical and Translational Science Center • University of Texas Health Science Center at San Antonio — UL1TR002645: Institute for Integration of Medicine and Science • Yale New Haven Hospital — UL1TR001863: Yale Center for Clinical Investigation
