# Supplementary figures and images for: Predicting Long COVID in the National COVID Cohort Collaborative Using Super Learner: Cohort Study
Source: JMIR Public Health Surveill. 2024 Aug 15;10:e53322. doi: 10.2196/53322 (PMC11364083; doi:10.2196/53322)

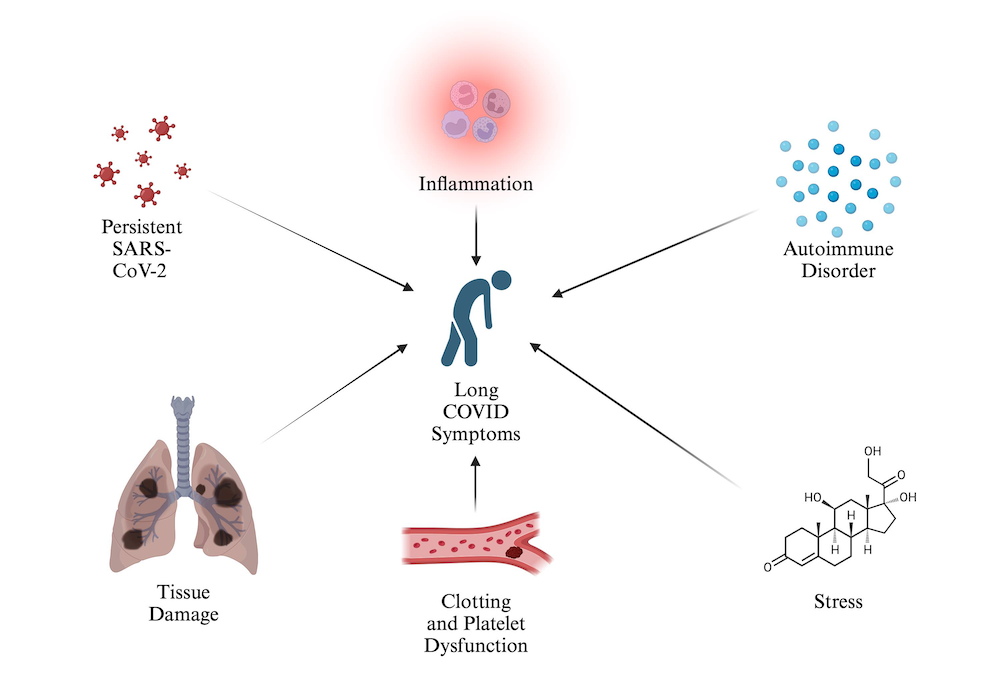

Supplement: Multimedia Appendix 1 [file publichealth_v10i1e53322_app1.png]
